# *Cecr2* mutant mice as a model for human cat eye syndrome

**DOI:** 10.1038/s41598-021-82556-y

**Published:** 2021-02-04

**Authors:** Renée Dicipulo, Kacie A. Norton, Nicholas A. Fairbridge, Yana Kibalnyk, Sabrina C. Fox, Lisa K. Hornberger, Heather E. McDermid

**Affiliations:** 1 Department of Biological Sciences, University of Alberta, CW 405 Biological Sciences Building, 11455 Saskatchewan Drive, Edmonton, AB T6G 2E9 Canada; 2 Office of Professional and Educational Development, Room 2973 Health Sciences Centre, Memorial University, St. John’s, NL A1B 3V6 Canada; 3 Division of Cardiology, Department of Pediatrics and Department of Obstetrics and Gynecology, Women’s and Children’s Health Research Institute, University of Alberta, Edmonton, Canada

**Keywords:** Genetics, Development, Medical genetics

## Abstract

Cat eye syndrome (CES), a human genetic disorder caused by the inverted duplication of a region on chromosome 22, has been known since the late 1890s. Despite the significant impact this disorder has on affected individuals, models for CES have not been produced due to the difficulty of effectively duplicating the corresponding chromosome region in an animal model. However, the study of phenotypes associated with individual genes in this region such as *CECR2* may shed light on the etiology of CES. In this study we have shown that deleterious loss of function mutations in mouse *Cecr2* effectively demonstrate many of the abnormal features present in human patients with CES, including coloboma and specific skeletal, kidney and heart defects. Beyond phenotypic analyses we have demonstrated the importance of utilizing multiple genetic backgrounds to study disease models, as we see major differences in penetrance of *Cecr2*-related abnormal phenotype between mouse strains, reminiscent of the variability in the human syndrome. These findings suggest that *Cecr2* is involved in the abnormal features of CES and that *Cecr2* mice can be used as a model system to study the wide range of phenotypes present in CES.

## Introduction

Cat eye syndrome (CES) is a rare human chromosomal disorder characterized by a highly variable phenotype^1^. The name comes from the eye defect coloboma, which results from the failure of the optic fissure to close during early eye development. This results in a permanent fissure in the iris, which often extends into the retina and can result in vision loss. However, coloboma can be bilateral or unilateral, and is only seen in 55–61% of CES patients^2,3^. Other features seen in CES include preauricular ear tags and pits (81–87%), heart defects (50–63%), kidney defects (31%), skeletal defects (29–73%), anal anomalies (73–81%), cleft palate (14–31%) and intellectual impairment (32–56%). The variability of the phenotype ranges from the presence of only minor dysmorphic features to the presence of multiple serious organ defects.

CES is the result of a triplication of a region of chromosome 22q11.2 in the form of a bisatellited supernumerary chromosome (inv dup22pter-22q11.2), known as the CES chromosome^4^. The extra material in the CES chromosome encompasses approximately 1.1 Mb adjacent to the pericentromeric region of chromosome 22^5^. The recurrent 22q11.2 breakpoints are each in one of two low-copy repeat regions, producing a smaller Type I or larger Type II CES chromosome^6^. Interestingly, the extra material in a Type II chromosome does not have a detectable effect on the phenotype^6^. The CES chromosome can be inherited, yet even in the same family the phenotype can be highly variable, leading to the possibility of a missed diagnosis in an affected parent until a child with cardinal CES features is born^7^.

Patients with CES can alternatively have an interstitial duplication of the same region of 22q11.2 (3 rather than 4 copies), and show a similar phenotype to those with the CES chromosome^8,9^. The rarity of such interstitial duplications and the general variability of the syndrome do not allow analysis of whether the presence of the full CES chromosome has a worse prognosis. There are no known cases of a reciprocal microdeletion of this region.

There are at least 14 RefSeq genes over the 1.1 Mb known to be present in the CES critical region based on the smaller type I CES chromosome^5,6^. The smallest region associated with the major features of CES was determined from a patient with all cardinal CES features and an unusually small supernumerary ring chromosome^10^, which eliminated the most distal 3 genes of the CES critical region (*BCL2L13*, *BID*, and *MICAL3*). A small 600 kb interstitial triplication has further narrowed the CES critical region for at least some features to 3 genes: *CECR2, SLC25A18* and *ATP6V1E1*^11^. This triplication was found in a three-generation pedigree and confirmed in 4 individuals. Associated symptoms were anal malformations, preauricular tags/pits, and renal anomalies. However, since the syndrome is so variable, it is not possible to extrapolate to whether other CES phenotypes may be associated with these genes.

There is no animal model currently available with the equivalent duplication of the CES critical region, but candidate gene mutations can provide insight into the function of these genes. Gene mutations in mice are usually loss of function, which would seem to be inappropriate for the study of a duplication syndrome. However, in humans the loss or gain of a chromosomal region has been shown to sometimes produce similar phenotypes. For instance, the loss or gain of one copy of a region of chromosome 22q11.2, adjacent to but not associated with the CES region, produces a microdeletion or microduplication syndrome with many similar symptoms to each other. In fact, the similarities to the 22q11.2 microdeletion syndrome (DiGeorge syndrome/velocardiofacial syndrome) led in part to the identification of the 22q11.2 microduplication syndrome^12^. One candidate gene for CES is *CECR2*, a gene in the original and modified CES critical regions. CECR2 is a chromatin remodelling protein and part of the CERF complex^13^. In mice, loss of CECR2 results in the misregulation of several mesenchymal/ectodermal transcription factors^14^, and this role in developmental regulation would support *CECR2* as a gene of interest in CES. *Cecr2* mutations in mice result in the lethal neural tube defect exencephaly, equivalent to human anencephaly^13^. Although neural tube defects have never been associated with CES, we hypothesized that there may be other more subtle features in this loss of function mouse that resemble CES clinical features and suggest the involvement of the gene in CES when duplicated.

Here we show that mice homozygous for mutations in *Cecr2* show coloboma, microphthalmia, and skeletal, heart, and kidney defects at variable penetrance in a strain specific manner, recapitulating many of the features of human CES associated with the gain of *CECR2* copies. This suggests the involvement of *CECR2* in multiple features of human CES, making the *Cecr2* mouse lines a useful model for understanding these features in CES.

## Materials and methods

### Mice

All experiments involving mice were approved by the Animal Care and Use Committee of the University of Alberta (University of Alberta AUP 00000094). All methods were performed in accordance with the relevant guidelines and regulations (Canadian Council on Animal Care). The mice were housed in individually ventilated cages (Tecniplast IVC blue line) with a 14 h light/10 h dark cycle and an ambient temperature of 22 ± 2 °C. Mice were fed ad libitum LabDiet Laboratory Rodent Diet 5001 except breeders, who were fed LabDiet Mouse Diet 9F 5020. Breeding females were housed with males and checked each morning for the presence of a copulatory plug, then separated and considered day 0.5 of pregnancy. Embryo ages are listed as E followed by the day of gestation.

### Mutations and strains

**Figure 1:**
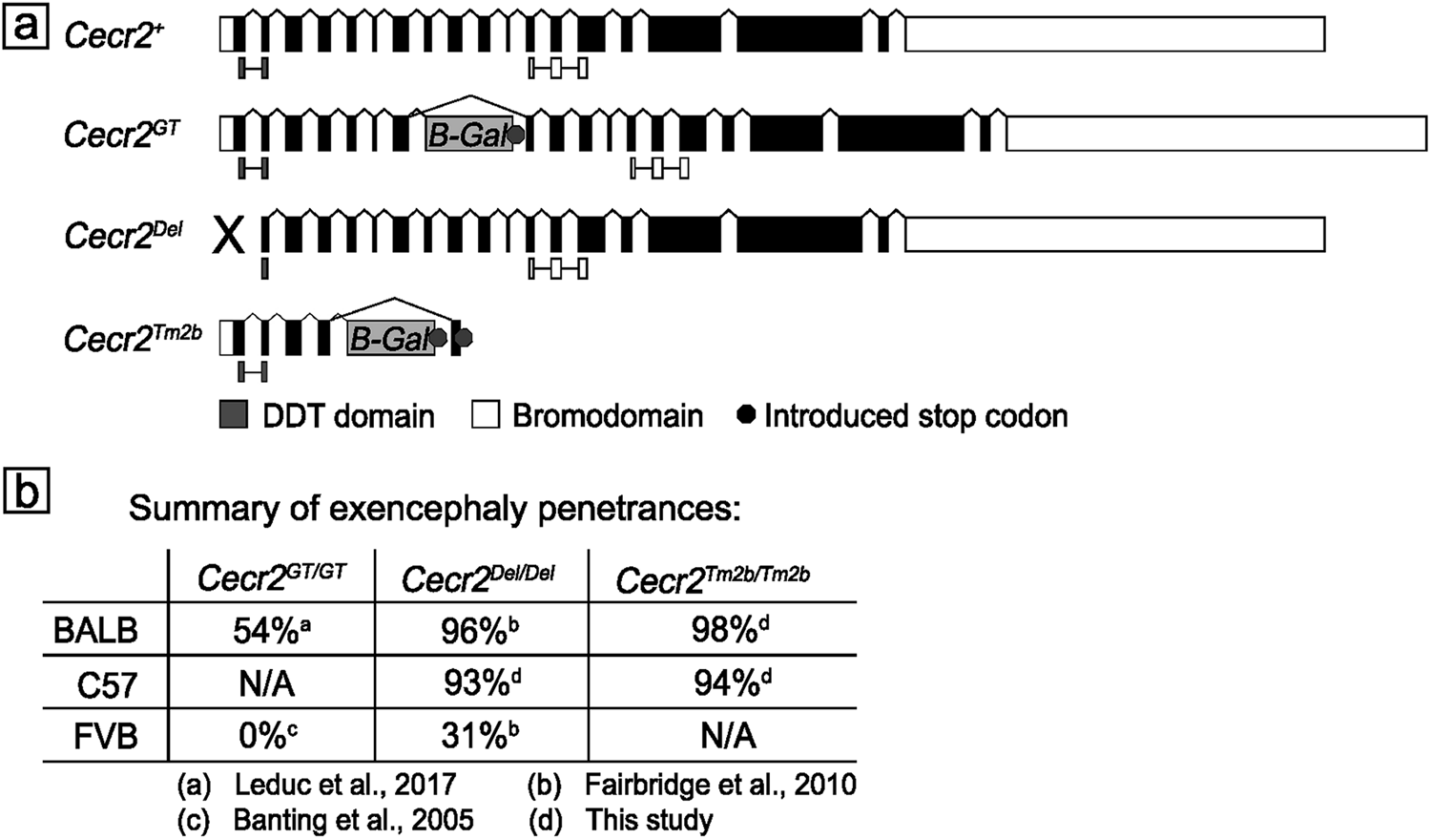
*Cecr2* mutations used in this study. Four alleles were used and the abbreviated notations of the 3 mutations used throughout this report are shown (**a**). The wildtype allele has 19 exons. A DDT domain is present in exons 1–2 and a bromodomain is located in exons 12–14. The *Cecr2*^*GT*^* (Cecr2*^*Gt(pGT1)1Hemc*^*)* genetrap allele results from a β-Gal construct inserted between exons 7 and 8, resulting in a CECR2/LacZ-fusion protein. However, the hypomorphic mutation is leaky and some full-length transcript is made, presumably by splicing around the genetrap. The presumptive null *Cecr2*^*Del*^* (Cecr2*^*tm.1.1Hemc*^*)* deletion allele is the result of deleting exon 1 and ~ 1 kb upstream. The presumptive null allele *Cecr2*^*tm2b*^* (Cecr2*^*tm2b(EUCOMM)Hmgu*^*)* is a β-Gal gene trap that produces a CECR2/LacZ-fusion protein, but also has *Cecr2* exon 4 excised, which puts all transcripts out of frame. A summary of the exencephaly penetrances of these mutations on different strain backgrounds (**b**) highlights the difference between the hypomorphic *Cecr2*^*GT*^ allele and the two presumptive null alleles and also the dramatic effect background strain has on the exencephaly phenotype.

This study used four different alleles of the *Cecr2* gene: the wild-type allele (*Cecr2*^+^), a genetrap which partially disrupts *Cecr2* (*Cecr2*^*Gt(pGT1)1Hemc*^ or *Cecr2*^*GT*^)^13^, a presumptive null deletion of the first exon (*Cecr2*^*tm.1.1Hemc*^ or *Cecr2*^*Del*^)^14^ and a second presumptive null genetrap (the *Cecr2*^*tm2b(EUCOMM)HMGU*^ with floxed exon 4 removed, or *Cecr2*^*tm2b*^) (Fig. 1a). In each case the simplified symbol is used in this report. These alleles were on three different backgrounds: C57Bl/6N, BALB/cCrlAlt and FVB/NJ. The BALB/cCrlAlt strain originated from Charles River Laboratories but was maintained independently since ~ 1988 at the University of Alberta and now differs in some respects from the original BALB/cCrl line. Each *Cecr2* mutation results in the lethal neural tube defect exencephaly at a variable penetrance, depending on the mutation and the strain (see Fig. 1b). The *Cecr2*^*GT*^ and *Cecr2*^*Del*^ alleles were congenic on BALB/cCrlAlt and FVB/NJ backgrounds. The *Cecr2*^*Del*^ allele was moved onto the C57Bl/6N background through successive matings to C57Bl/6N wild types and analyzed from generation 3 to 5 (N3–N5). The *Cecr2*^*tm2b*^ allele was congenic on a C57Bl/6N background and was moved to BALB/cCrlAlt background and analyzed from N4–N5.

### Histology

Tissues and embryos were fixed in 10% formalin immediately upon dissection. Embryos were cut at the neck and abdomen to allow fixative to enter. Eyes, hearts and kidneys were later dissected from the embryos. Tissues were processed and embedded in paraffin, serially sectioned at 5 or 7 μm and stained in hematoxylin and eosin (H&E). X-Gal staining was done on embryos and isolated kidneys as previously described in Banting et al. 2005^13^. For immunostaining, antigen retrieval was done on paraffin sections using 95 °C 1 mM EDTA (pH 8), then sections were blocked in 10% normal goat serum for 2 h. They were then incubated overnight at 4 °C with 1:20,000 anti-CECR2 antibody^15^, followed by AlexaFluor-488 goat anti-rabbit secondary antibody (Life Technologies) at 1:200 for 2 h at room temperature. All rinses were done with PBS at 2 × concentration. After counterstaining with DAPI, Fluoromount-G was used for mounting and imaging was accomplished using a Nikon Eclipse 80i confocal microscope.

### Analysis of cartilage and bone in developing embryo

Alcian blue and alizarin red whole mount skeletal staining was performed on E17.5–E18.5 embryos as described by^16^. In brief, embryos were dissected from the uterus and scalded at 65℃. Embryos were then skinned and washed in 95% ethanol. Embryos were stained for at least 24 h in Alcian Blue stain, rinsed, and cleared with 1% KOH solution. After clearing, embryos were counterstained in 0.005% Alizarin Red in 2% KOH. Images were obtained by immersing embryos in 1:1 glycerol and 2% KOH in agar coated dishes, and then photographed.

### Heart casting

Heart resin casts were produced from E17.5–18.5 embryos using the Batson’s No. 17 Plastic Replica and Corrosion Kit (Polysciences, Inc) prepared according to manufacturer instructions. Embryos were dissected from the uterus and decapitated to allow flow of the resin through the heart. The pericardial cavity was exposed and a 1 cc insulin syringe was used to inject resin into the right, then left ventricle for each embryo. The embryo body was left on ice for 3 h to overnight in a refrigerator to allow the resin to harden. Tissue was then removed from the cast through immersion in Maceration Fluid from several hours to overnight, followed by washing in distilled water. Casts were dissected to reveal the pulmonary arteries and veins on the dorsal side of the heart, then photographed using an Olympus SZ61 microscope and SeBaCam5C camera. Casts were injected with blue-dyed resin in the right ventricle and red-dyed resin in the left ventricle, but the dyes mixed unpredictably due to the presence of the foramen ovale connecting the atria. Because of this, meaningless colour variation in the vessels can be seen even in the greyscale images shown.

### Micro-CT imaging

Mouse tails were fixed in Bouin’s fixative for up to one week and incrementally washed into 100% ethanol. The skeletal structures were serially X-rayed in a Skyscan 1076 at 35 μm resolution. Computer tomography rendered the X-rays into a 3D skeletal model using programs CTAn, CTVol, CTVox, and DataViewer. The rendered models were visually inspected for skeletal defects.

### Statistical analysis

Phenotype penetrance was compared using a two-tailed Fisher’s Exact test.

## Results

### Cecr2 mutant mouse embryos show coloboma and other eye defects

The hallmark feature of CES is the eye defect coloboma, where the inferior choroid fissure in the eye fails to close, resulting in a gap in tissues such as the retina, cornea, and iris^17^. While CES is named after the coloboma that presents in the majority of patients (55–61%), other features such as microphthalmia may present as well^2,3^.

**Figure 2:**
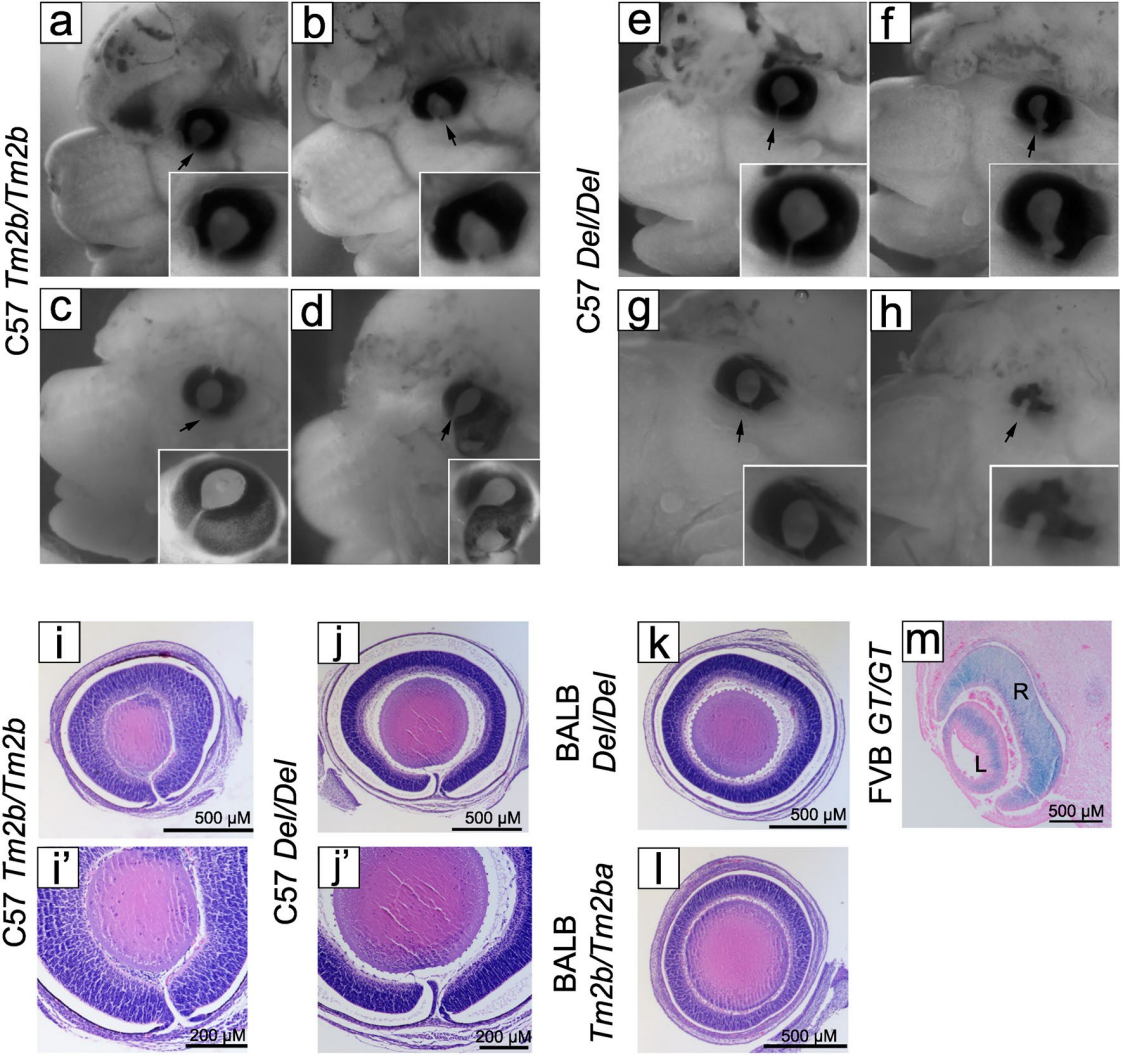
Loss of CECR2 results in coloboma and other eye defects. Examples of colobomas in *Cecr2*^*Tm2b/Tm2b*^ (**a**–**d**) and *Cecr2*^*Del/Del*^ (**e**–**h**) embryos on a C57Bl/6N background show the variation in the severity of the defects. Each image has an inset at higher magnification. In (**c**) and (**d**) insets the eye was photographed after removal. The additional defect of microphthalmia is seen in (**h**). For the 2 mutations on a BALB/cCrlAlt background, which have unpigmented eyes, identification of the presence of coloboma was determined partially or completely by histology. Eyes were serially sectioned in the frontal plane and H&E stained. Colobomas were present on a C57Bl/6N background (**i**,**j**, with **i’** and **j’** at higher magnification) but not on a BALB/cCrlAlt background (**k**,**l**). X-gal staining of an eye from an FVB/NJ *Cecr2*^*GT/GT*^ embryo at E12.5 suggested that *Cecr2* is expressed in the forming retina and lens around the time of optic fissure closure. L = lens, R = forming retina. All embryos pictured also had exencephaly.

The phenotypic effects of a new *Cecr2* mutation *Cecr2*^*Tm2b*^ were examined in embryos on a C57Bl/6N background during late gestation from E15.5 to E18.5. The penetrance of exencephaly in this line was 94% (68/72, Table 1), so eyes were examined before birth. In mice, the choroid fissure begins to close at early E12, and is fully closed by late E12^18^. Therefore, E15.5–E18.5 is well after the choroid fissure should be fully closed. We found that a high percentage of *Cecr2*^*Tm2b/Tm2b*^ embryos showed coloboma in a single or both eyes (Fig. 2a–d). Out of 72 *Cecr2*^*Tm2b/Tm2b*^ embryos, 59/72 (82%) had coloboma (Table 1), with 41/59 (69%) being bilateral and 18/59 (31%) being unilateral (Table 2). Not all colobomas were uniform, and several variations in appearance were observed (Fig. 2a–d). Most colobomas varied in the degree of separation in the fissure (Fig. 2a–c). In a single case, we found a protrusion on the inferior side of the eye (Fig. 2d). Microphthalmia, a condition where the eye develops abnormally small, is observed in 19–39% of CES patients^2,3^. When examining *Cecr2*^*Tm2b/Tm2b*^ embryos, we observed 6 with microphthalmia (6/72, 8.3%) that presented in embryos that also had coloboma (Table 1). Microphthalmia was not observed independently of coloboma (Table 2). No embryos with coloboma (*p* < 0.0001) or microphthalmia (0.0057) were observed in wild-type embryos (0/95, 0%, Table 1). We also analyzed *Cecr2*^*Tm2b/*+^ heterozygous embryos. We found 1 with coloboma out 197 *Cecr2*^*Tm2b/*+^ embryos (1/197, 0.5%, *p* > 0.05 compared to wildtype, Table 1). In this single coloboma in a *Cecr2*^*Tm2b/*+^ embryo, there was no exencephaly observed. No embryos with microphthalmia were observed in the *Cecr2*^*Tm2b/*+^ embryos (0/197, 0%, Table 1).

**Table 1:** Penetrance of defects in late stage embryos, with 3 different *Cecr2* mutations on 3 different genetic backgrounds.

Background	Genotype	Exencephaly^a^	Coloboma	Microphthalmia	Polydactyly	Heart VSD defect	Heart PV defect	Duplex kidney^b^
C57Bl/6J	*Cecr2*^Tm2b/Tm2b^	68/72 (94%)	59/72 (82%)	6/72 (8.3%)	36/72 (50%)	5/22 (23%)^c^	5/7 (71%)^d^	1/30 (3.3%)
	*Cecr2*^Tm2b/^^+^	1/197 (0.5%)	1/197 (0.5%)	0/197 (0%)	14/197 (7.1%)	ND	ND	ND
	*Cecr2*^+/+^	0/95 (0%)	0/95 (0%)	0/95 (0%)	0/95 (0%)	0/22 (0%)	0/7 (0%)	ND
	*Cecr2*^Del/Del^	27/29 (93%)	17/29 (59%)	3/29 (10%)	12/29 (41%)	ND	2/2 (100%)	ND
	*Cecr2*^Del/^^**+**^	2/76 (2.6%)	1/76 (1.3%)	1/76 (1.3%)	1/76 (1.3%)	ND	ND	ND
	*Cecr2*^+/+^	0/29 (0%)	0 /29 (0%)	0/29 (0%)	0/29 (0%)	ND	ND	ND
BALB/cCrlAlt	*Cecr2*^Tm2b/Tm2b^	47/48 (98%)	0/27 (0%)	0/27 (0%)	32/48 (67%)	0/26 (0%)	3/3 (100%)	0/27 (0%)^e^
	*Cecr2*^Tm2b/^^+^	1/131 (0.8%)	0/48 (0%)	0/48 (0%)	13/131 (9.9%)	ND	ND	ND
	*Cecr2*^+/+^	0/70	0/2 (0%)	0/2 (0%)	0/70	0/9 (0%)	0/2 (0%)	ND
	*Cecr2*^Del/Del^	96%^g^	0/7 (0%)	0/7 (0%)	11/15 (73%)	ND	3/3 (100%)	ND
	*Cecr2*^**Del/**^^**+**^	ND	ND	ND	ND	ND	ND	ND
	*Cecr2*^+/+^	0%^g^	0/2 (0%)	0/2 (0%)	ND	ND	0/1 (0%)	ND
FVB	*Cecr2*^*GT/GT*^	0%^h^	ND	ND	ND	ND	ND	8/20 (40%)^f^
	*Cecr2*^*Del/Del*^	31%^g^	ND	ND	ND	ND	ND	13/27 (48%)
	*Cecr2*^+/+^	0%^h^	ND	ND	ND	ND	ND	0/9 (0%)

Numbers represents the number of embryos (ND = not done).

^a^Exencephaly penetrance applies only to the coloboma/micropthalmia/polydactyly datasets.

^b^At least one duplex kidney present in the embryo.

^c^In addition 2/22 showed hypoplastic RV, which was not seen in wildtype controls.

^d^1/7 showed a defect of the great vessels, and 2/7 showed abnormal vessels from the right lung to the RSVC.

^e^1/27 had one kidney with a central cavity which may have been hydronephrotic and duplex.

^f^168 adults were also examined: 16 (9.5%) had unilateral agenesis, 3 (1.7%) had hydronephrosis.

^g^Fairbridge et al., 2010.

^h^Banting et al., 2005.

**Table 2:** Penetrance of polydactyly and eye defects in detail of the *Cecr2 Tm2b* and *Cecr2 Del* mutations on 2 genetic backgrounds.

Background	Genotype	Pre-axial polydactyly	Post-axial polydactyly	Both	Unilateral coloboma	Bilateral coloboma	Microphthalmia with coloboma	Microphthalmia without coloboma
C57Bl/6J	*Cecr2*^Tm2b/Tm2b^	27/36 (4/30 bilateral)	6/36 (3/9 bilateral)	3/36	18/59	41/59	6/6	0/6
	*Cecr2*^Tm2b/+^	5/14 (2/5 bilateral)	9/14	0/14	1/1	0/1		
	*Cecr2*^Del/Del^	11/12 (1/11 bilateral)	1/12 (1/11 bilateral)	0/12	3/17	14/17	3/3	0/3
	*Cecr2*^Del/+^	0/1	1/1	0/1	1	0/1		
BALB/cCrlAlt	*Cecr2*^Tm2b/Tm2b^	0/32	32/32 (4/32 bilateral)	0/32				
	*Cecr2*^Tm2b/+^	0/30	13/30 (2/13 bilateral)	0/13				
	*Cecr2*^Del/Del^	1/11	11/11	1/11				
	*Cecr2*^Del/+^	ND	ND	ND				

To assess whether there is a difference in phenotypic severity between the *Cecr2*^*Tm2b*^ and *Cecr2*^*Del*^ mutations, we observed the *Cecr2*^*Del*^ mutation in the C57Bl/6 N background as well. In *Cecr2*^*Del/Del*^ embryos we noted 17/29 embryos with coloboma (17/29, 59%, Table 1), which is significantly lower than *Cecr2*^*Tm2b/Tm2*^ embryos (*p* = 0.0212). Similar to C57Bl/6 N *Cecr2*^*Tm2b/Tm2b*^ embryos, we saw a range of coloboma severity in *Cecr2*^*Del/Del*^ embryos (Fig. 2e–h). Within these 17 embryos with coloboma, 14 were bilateral (14/17, 82%, Table 2). In addition, 3 embryos with microphthalmia presenting with coloboma were observed (3/29, 10%, Tables 1, 2, Fig. 2h). Microphthalmia was not observed independently of coloboma (Table 2). We also analyzed *Cecr2*^*Del/*+^ heterozygotes. We found 1 embryo with coloboma, which also showed microphthalmia, in 76 *Cecr2*^*Del/*+^ embryos (1/76, 1.3%) (Table 1). Unlike *Cecr2*^*Del/Del*^, no embryos with coloboma (*p* < 0.0001) or microphthalmia (*p* > 0.05) were seen in *Cecr2*^+*/*+^ littermates (0/29, 0%, Table 1).

To observe colobomas at a more detailed resolution, C57Bl/6N embryo eyes with either the *Cecr2*^*Del*^ or *Cecr2*^*Tm2b*^ mutation were examined via histology at E18.5 (Fig. 2i–j’). For both mutations the coloboma fissure was clearly visible on the inferior aspect of the eye.

To determine whether genetic background affects coloboma penetrance, we examined both the *Cecr2*^*Del*^ and *Cecr2*^*Tm2b*^ mutations in the BALB/cCrlAlt background. BALB/cCrlAlt mice are albino and therefore lack pigment in the eyes, making coloboma very difficult to identify by examining the whole eye externally. We therefore examined BALB/cCrlAlt *Cecr2*^*Del/Del*^ embryonic eyes histologically. Out of 7 embryos that we sectioned and stained, 0 had coloboma (0/7, 0%, Table 1, Fig. 2k). No BALB/cCrlAlt *Cecr2*^+*/*+^ littermates sectioned had coloboma (0/2, 0%, Table 1). Similarly, we assayed BALB/cCrlAlt *Cecr2*^*Tm2b/Tm2b*^ embryonic eyes using histology. However, while crossing the *Cecr2*^*Tm2b*^ mutation from C57Bl/6N into the BALB/cCrlAlt background, we selected to maintain the brown colouration, allowing potential colobomas to be visualized in the whole eye without the need for histology in some cases. Combining histological analyses and visual analyses, we found that 0/27 BALB/cCrlAlt *Cecr2*^*Tm2b/Tm2b*^ embryos had coloboma (0/27, 0%, Table 1, Fig. 2l). Of these 27 samples, 5 were analyzed via histology and 22 were observed from dissected embryos. Microphthalmia was not observed in BALB/cCrlAlt *Cecr2*^*Tm2b/Tm2b*^ embryos. In *Cecr2*^*Tm2b/*+^ heterozygotes with brown eyes none were seen with coloboma or microphthalmia (0/48, 0%, Table 1). In BALB/cCrlAlt wild-type embryos, no eyes that were sectioned had coloboma or microphthalmia (0/2, 0%, Table 1). Fisher’s exact tests showed a significant difference between the C57Bl/6N and BALB/cCrlAlt backgrounds for both the *Cecr2*^*Tm2b*^ (*p* < 0.0001) and *Cecr2*^*Del*^ (*p* = 0.0080) mutations.

These results strongly indicate that the presence of coloboma in *Cecr2* mutant mice is strain dependent, being present on C57Bl/6N but not BALB/cCrlAlt backgrounds with both *Cecr2* mutations tested.

Furthermore, X-gal staining of an FVB/NJ *Cecr2*^*GT/GT*^ embryo at E12.5 suggested that *Cecr2* is expressed in the forming retina and lens around the time of optic fissure closure (Fig. 2m).

### *Cecr2* mutant embryos show skeletal defects

CES is also characterized by skeletal defects. Previous work in our lab has shown that *Cecr2* is expressed in the developing limbs^13^. Specifically, a CECR2/LacZ-fusion protein was seen in the skeletal structure of the fore and hind limbs and this expression resolved to the fore and hind paws later in development. However, no structural abnormalities were seen in the limbs of *Cecr2*^*GT/GT*^ embryos. Nevertheless, we analyzed the *Cecr2*^*Tm2b/Tm2b*^ and *Cecr2*^*Del/Del*^ embryos on both C57Bl/6N and BALB/cCrlAlt backgrounds during late gestation (E15.5–E18.5) for limb abnormalities.

**Figure 3:**
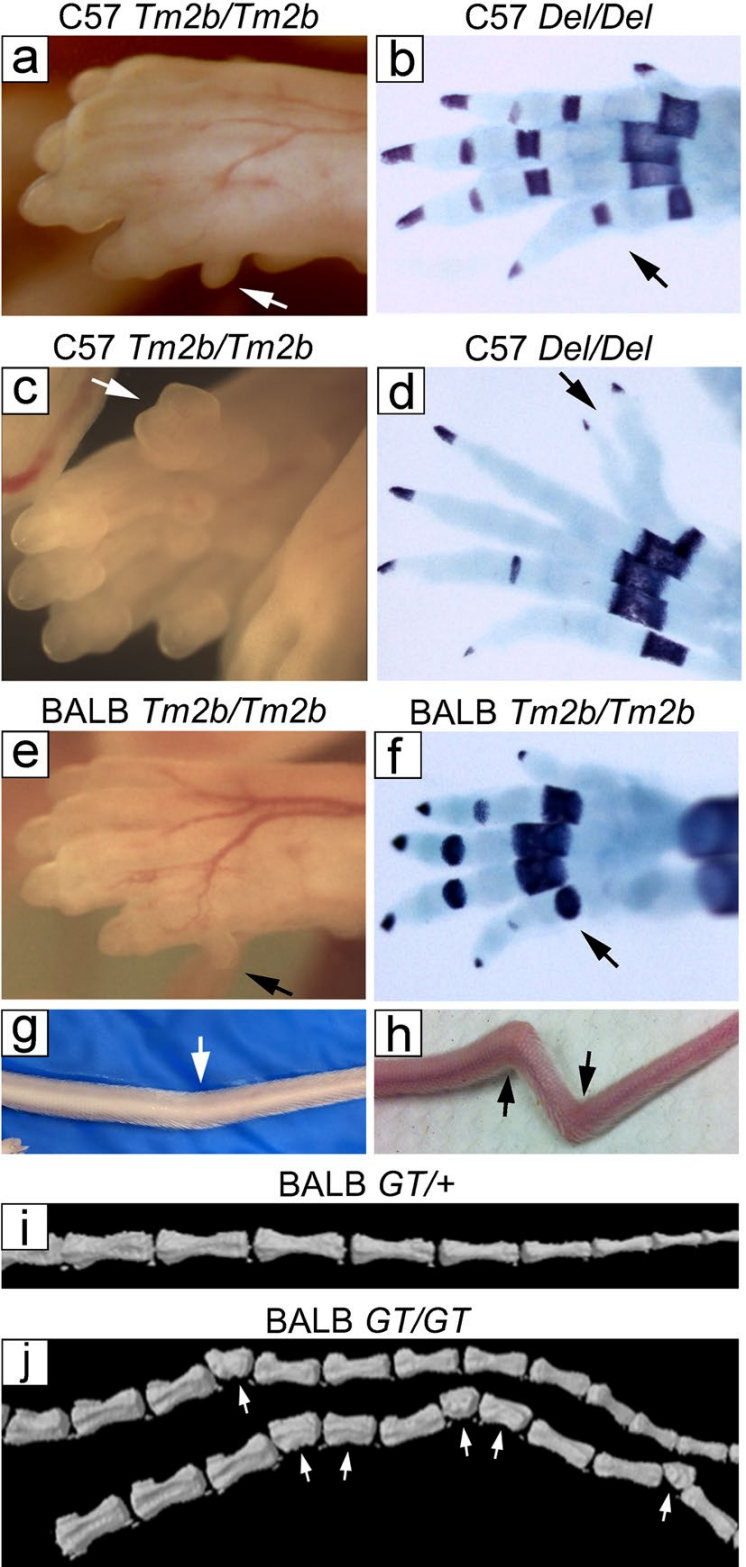
Loss of CECR2 results in skeletal defects. Both post-axial polydactyly of the forelimb and pre-axial polydactyly of the hindlimb were seen in *Cecr2*^*Tm2b/Tm2b*^ embryos on a C57Bl/6N background (**a**,**c** respectively), whereas only post-axial polydactyly of the forelimb was seen in *Cecr2*^*Tm2b/Tm2b*^ embryos on a BALB/cCrlAlt background (**e**). Representative limbs, with defects similar to those pictured in (**a**) (**c**) and (**e**), were stained with Alcian blue and Alizarin red to identify forming cartilage (light blue) and bone (dark staining) respectively (**b**,**d**,**f**). Post-axial extra digits did not contain cartilage or bone, but pre-axial extra digits did, formed by bifurcation of the first normal digit (**c**,**d**). BALB/cCrlAlt *Cecr2*^*GT/GT*^ adults also can show skeletal defects of the tail (arrows), with minor (**g**) and severe kinks (**h**). Compared to a heterozygote with no tail kinks (**i**), micro-CT scans of two mutant tails (**j**) showed intermittent shortened, wedge or hemi-vertebrae (arrows).

We observed 2 major polydactyly phenotypes in E15.5–18.5 embryos. Mice have 5 digits on both their fore- and hindlimbs. Post-axial polydactyly manifested as a small piece of tissue protruding from the ulnar side of the forelimb (Fig. 3a,e). This post-axial protrusion was never seen on the hindlimb. Limbs that were observed to have this phenotype were then assayed with Alcian blue and Alizarin red for cartilaginous or skeletal changes. Limbs with post-axial polydactyly showed no underlying cartilaginous or skeletal changes (Fig. 3b,f).

The other polydactyly phenotype was pre-axial polydactyly, where the extra digit was on the tibial side of the hindlimb, and was never seen on the forelimb. The outermost/first normal digit appeared bifurcated, resulting in an extra digit (Fig. 3c,d). When stained with Alcian blue and Alizarin red, the pre-axial digit was shown to have a cartilaginous component that split to create two digits with bone components on the end of each (Fig. 3e,f).

In the C57Bl/6N background with the *Cecr2*^*Tm2b*^ mutation, we observed both post- and pre-axial polydactyly (Fig. 3c–f, Tables 1 and 2). In total, 36/72 incidences of polydactyly were observed in *Cecr2*^*Tm2b/Tm2b*^ embryos (50%). Pre-axial polydactyly was found in 27/72 *Cecr2*^*Tm2b/Tm2b*^ embryos (38%), while 6/72 *Cecr2*^*Tm2b/Tm2b*^ embryos had post-axial polydactyly (8.3%) and 3/72 *Cecr2*^*Tm2b/Tm2b*^ embryos had both (4.2%). *Cecr2*^*Tm2b/*+^ heterozygotes had 14/197 embryos with polydactyly (7.1%,), with 5/197 pre-axial (2.5%), 9/197 post-axial (4.6%) and 0/197 with both. In the BALB/cCrlAlt background with the *Cecr2*^*Tm2b*^ mutation, only post-axial polydactyly was observed (Fig. 3a). In *Cecr2*^*Tm2b/Tm2b*^ embryos 32/48 showed polydactyly (67%). We also observed post-axial polydactyly in 13/131 *Cecr2*^*Tm2b/*+^ heterozygotes (9.9%).

The *Cecr2*^*Del*^ mutation on both the BALB/cCrlAlt and C57Bl/6N backgrounds showed similar results, although fewer embryos were examined (Tables 1, 2). C57Bl/6 N *Cecr2*^*Del/Del*^ embryos showed 12/29 incidences of polydactyly (41%), with 1/29 being post-axial (3.4%), 11/29 pre-axial (38%) and 0/29 with both. Only 1 case of post-axial polydactyly was seen in 76 *Cecr2*^*Tm2b/*+^ heterozygotes examined (1.3%). *Cecr2*^*Del/Del*^ embryos on the BALB/cCrlAlt background showed 11/15 embryos with polydactyly (73%), with 10/15 being post-axial only (67%), 0/15 being pre-axial only, and 1/15 showing both (6.7%). Overall, there was no significant difference in penetrance of the polydactyly phenotype between homozygotes with the *Cecr2*^*Tm2b*^ and *Cecr2*^*Del*^ mutations in either the C57Bl/6N or BALB/cCrlAlt background (*p* > 0.05 for all 4 comparisons).

BALB/cCrlAlt *Cecr2*^*GT/GT*^ and *Cecr2*^*Del/Del*^ mice also have tail kinks, a defect commonly associated with neural tube defects. Tail kinks ranged in severity from a single small kink to multiple large kinks (Fig. 3g,h respectively). Tail kinks are common in mutants, and were quantified at 37/72 (51%) in a study of BALB/cCrlAlt *Cecr2*^*GT/Del*^ compound heterozygotes. They were also seen at a lower penetrance in heterozygotes (although not quantified), but were never or rarely seen in BALB/cCrlAlt wildtype mice. Micro-CT imaging was used to examine the tails of a *Cecr2*^*GT/*+^ heterozygote without tail kinks and 2 homozygotes with tail kinks (Fig. 3i,j, respectively). The homozygotes showed occasional shortened, wedge and hemi-vertebrae at one or multiple sites along the tail. We did not examine the upper spine for vertebral defects.

### *Cecr2* mutant mouse embryos show multiple heart defects

CES is characterized by heart defects in 50–63% of human patients^2,3^. Common abnormalities include septal defects, both ventricular (VSDs, 36%) and atrial (ASDs, 30%)^3^. Total anomalous pulmonary venous return (TAPVR), occurring in 30–43% of patients^2,3^, is a defect of the patterning of the pulmonary veins such that they drain directly or indirectly into the right rather than left atrium. To see if mice with a loss of CECR2 have similar heart defects, we examined E15.5–18.5 mutant and wild-type embryos using serial sections to detect VSDs and embryonic heart resin casting in E17.5–18.5 embryos to detect abnormalities of the pulmonary veins (Table 1).

**Figure 4:**
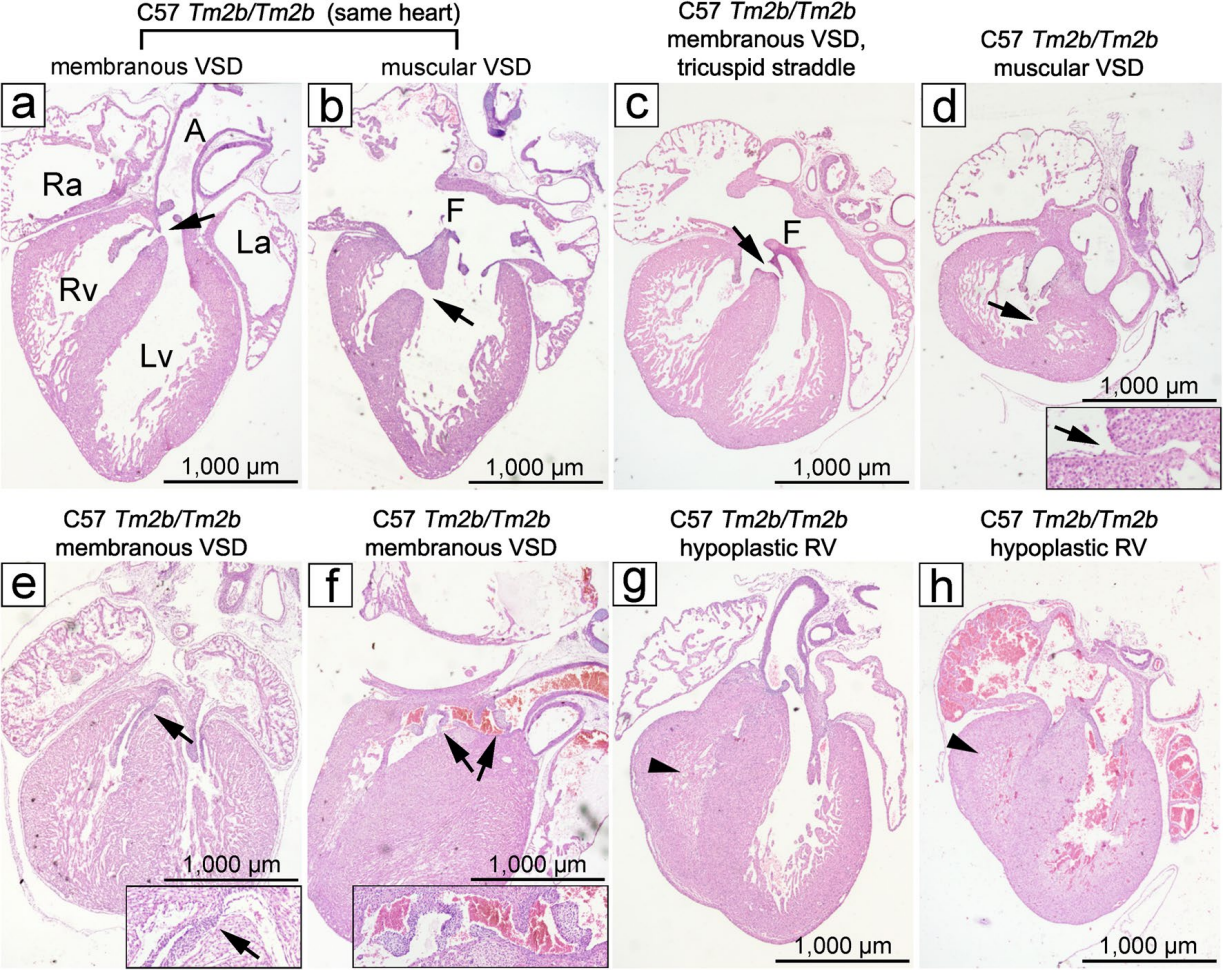
Loss of CECR2 results in VSDs. Defects were seen in coronally serial sectioned hearts of 5/22 *Cecr2*^*Tm2b/Tm2b*^ embryos E15.5–E18.5 embryos. One embryo (**a**,**b**) had both a membranous and muscular VSD (arrows). Four other embryos showed VSDs, one membranous VSD with a tricuspid straddle (**c**), one small muscular VSD (**d**) and two small membranous VSDs (**e**,**f**). Two embryos also showed hypoplastic right ventricles (RV) (arrowheads), appearing small throughout all serial sections rather than reflecting sectioning at an angle to the coronal plane (**g**,**h**). H&E staining. Heart images were taken at 2.5 × magnification, with insets at 10 × magnification. A-Aorta, La-left atrium, Lv-left ventricle, Ra-right atrium, Rv-right ventricle, F- foramen ovale.

Serial sections from 22 *Cecr2*^*Tm2b/Tm2b*^ embryos on a C57Bl/6 N background revealed 5 embryos with VSDs (23%), compared to 0/22 VSDs in wild-type littermates (*p* = 0.0485). Two embryos had large defects: one had both membranous and muscular VSDs (Fig. 4a,b) while another had a membranous VSD with a tricuspid straddle (Fig. 4c). Three additional embryos had small VSDs, 1 muscular (Fig. 4d) and 2 membranous (Fig. 4e,f). Furthermore, 2/22 mutants (9%) had a hypoplastic right ventricle (RV) (Fig. 4g,h), compared to 0/22 in wild-type littermates, although this observation did not reach statistical significance (*p* > 0.05). The presence of VSDs was strain dependent. On a BALB/cCrlAlt background, no heart defects were seen in 26 *Cecr2*^*Tm2b/Tm2b*^ embryos (*p* = 0.0154 compared to a C57Bl/6 N background) or 9 wild-type littermates. Hypoplastic RV was also not seen on the BALB/cCrlAlt background.

**Figure 5:**
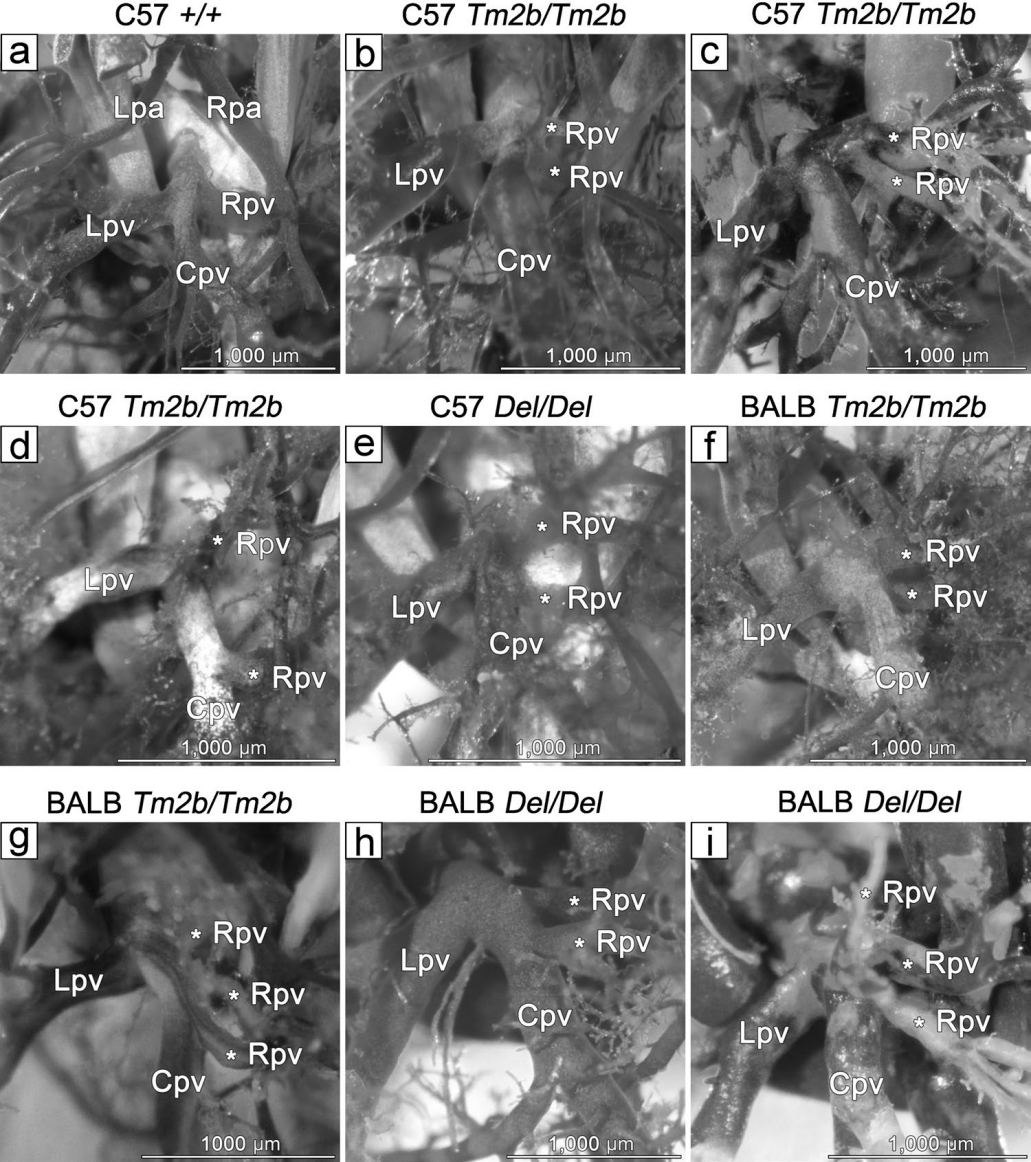
Loss of CECR2 results in abnormal patterning of the right pulmonary vein (RPV). Representative pictures of the PVs are shown for different mutations and genetic backgrounds. The asterisks label abnormal RPVs, either 2 (**a**–**f**,**h**) or 3 (**g**,**i**), as opposed to the normal single RPV in a control (**a**). Casts were made by injecting resin into the hearts of E17.5–18.5 embryos and then dissecting away extraneous tissues and vessels to reveal the dorsal aspect of the heart. Cpv-central pulmonary vein, Lpa-Left pulmonary artery, Lpv-left pulmonary vein, Rpa-right pulmonary artery, Rpv-right pulmonary vein.

In order to determine if there was aberrant patterning of the major blood vessels of the heart, and particularly the pulmonary veins (PV), we examined the resin casting of E17.5–E18.5 hearts (Table 1). The majority of mutant embryos showed a subtle abnormality of the PV organization, suggesting that *Cecr2* is involved in PV patterning. Mice normally have 3 PVs (left, right and central, or alternatively left, right inferior and right superior) that join in a duct to the left atrium^19^ (Fig. 5a), unlike humans who have 2 left and 2 right PVs. In mice the central vein then branches and drains both lungs (although primarily the right). On the C57Bl/6N background 5/7 *Cecr2*^*Tm2b/Tm2b*^ embryos had 2 smaller right PVs (RPVs), which branched either at or near the duct (Fig. 5b,c), or a small RPV with a novel rightward branch from the central PV (Fig. 5d). The patterning of PVs in wild-type littermates was normal in 7/7 embryos (*p* = 0.021). Interestingly, 1 of the mutants with 2 RPVs (Fig. 5b) did not have exencephaly (a rare occurrence in mutants), which suggests that the aberrant PV patterning was not an indirect consequence of the neural tube defect. We also examined 2 C57Bl/6N *Cecr2*^*Del/Del*^ embryos, both of which had 2 RPVs (Fig. 5e). Preliminary evidence indicates that abnormal RPV patterning is also typical for both mutations on a BALB/cCrlAlt background. Three mutants for each mutation were examined, and for both *Cecr2*^*Tm2b/Tm2b*^ and *Cecr2*^*Del/Del*^ embryos, 2 had 2 RVs and 1 had 3 (Fig. 5f–i). The 3 *Cecr2*^+*/*+^ wild-type littermates examined had normal PV patterning.

**Figure 6:**
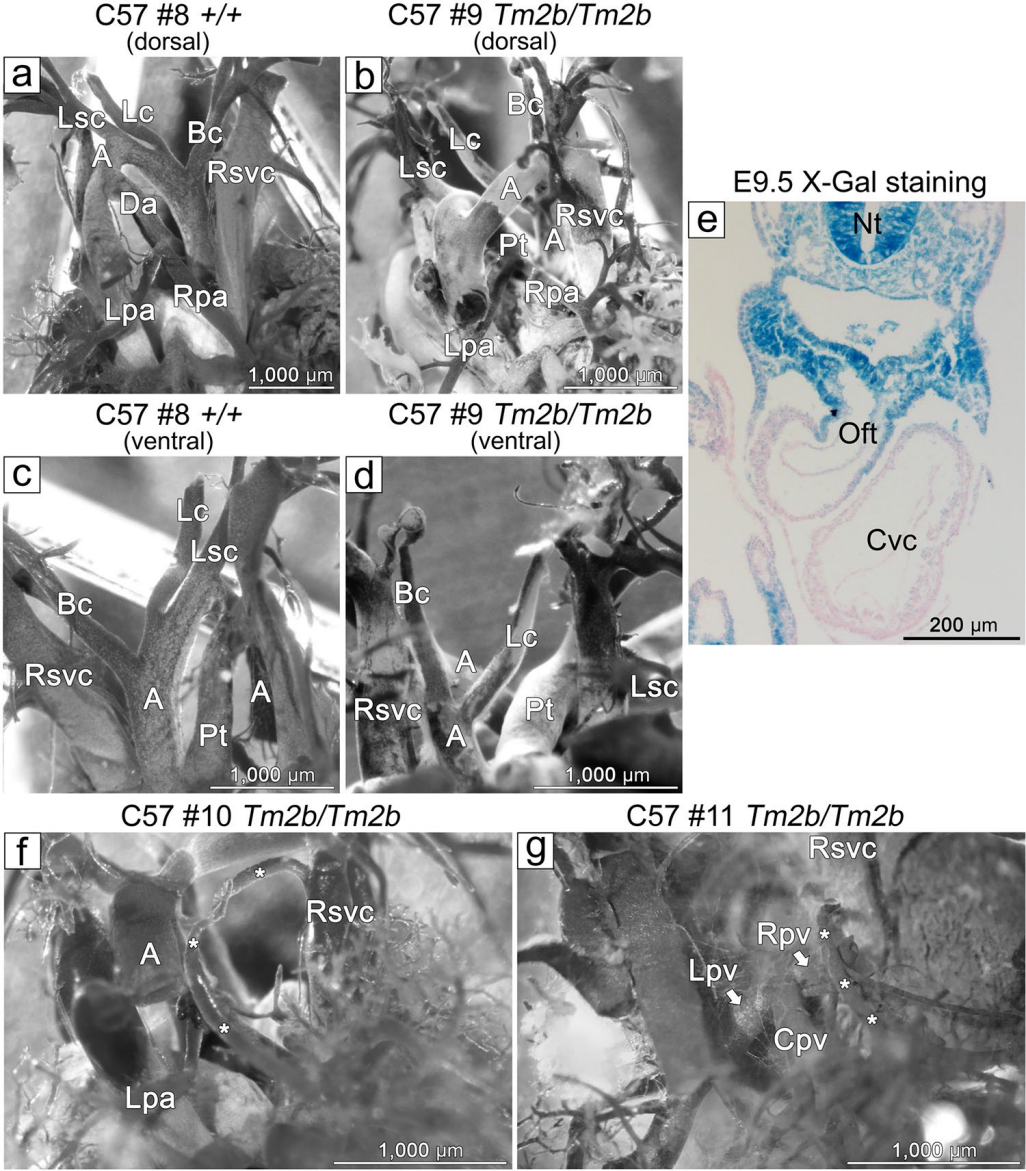
Other heart defects resulting from loss of CECR2. Embryonic resin heart casts revealed that 1/7 C57Bl/6N *Cecr2*^*Tm2b/Tm2b*^ embryos (**b** dorsal, **d** ventral) showed what appears to be right aortic arch with isolated left subclavian artery (Lsc) compared to a normal embryo (**a** dorsal, **c** ventral). The Lsc arises from the pulmonary tract/ductus arteriosus rather than the aorta. X-gal staining of a *Cecr2*^*GT/GT*^ E9.5 embryo (**e**) shows *Cecr2* expression in the outflow tract (Oft) of the heart, which becomes the ascending aorta and pulmonary tract. In 2/7 C57Bl/6N *Cecr2*^*Tm2b/Tm2b*^ embryos (**f**,**g**), an aberrant vessel (marked along the length with asterisks) was seen coming from the right lung and draining into the right superior vena cava. A-aorta, Bc-brachiocephalic artery, Cpv-central pulmonary vein, Cvc-common ventricular chamber, Da-ductus arteriosus, Lc-left carotid artery, Lpa-left pulmonary artery, Lpv-left pulmonary vein, Lsc-left subclavian artery, Nt-neural tube, Oft-outflow tract, Pt-pulmonary tract, Rpa-right pulmonary artery, Rsvc-right superior vena cava.

While examining PV patterning, we also saw other vessel abnormalities. An unusual abnormality of the great vessels was seen in 1 C57Bl/6 *Cecr2*^*Tm2b/Tm2b*^ embryo (Fig. 6b,d) compared to a control littermate (Fig. 6a,c). The placement of the great vessels suggests a right aortic arch with isolated origin of the left subclavian artery, where the latter appeared to originate from the pulmonary trunk or ductus arteriosus rather than the aortic arch. X-gal staining of E9.5 embryos showed *Cecr2* expression in the outflow tract of the heart, which becomes the ascending aorta and pulmonary trunk, but little expression in the rest of the heart (Fig. 6e). In addition, 2/7 C57Bl/6 *Cecr2*^*Tm2b/Tm2b*^ embryos had an abnormal vessel coming from the right lung and draining into the right superior vena cava (RSVC) rather than the RPV (Fig. 6f,g). One of these (Fig. 6f) was the mutant that did not have exencephaly. A vessel draining from the lung into the RSVC is reminiscent of defects seen in human partial anomalous pulmonary venous return, where 1 or 2 of the PVs drain into the left atrium or dorsal vena cava^20^. None of these defects were seen in any wild-type embryo heart casts.

### *Cecr2 *mutant mouse embryos and adults show kidney defects

Kidney defects are a common feature of human CES, particularly unilateral renal agenesis (38%, 29/77 kidney defects) and hydronephrosis (34%, 26/77 kidney defects)^2,3^. Duplex kidney has also been reported^21,22^. We found all 3 defects in *Cecr2* homozygous mice on the FVB/NJ background (Table 1).

**Figure 7:**
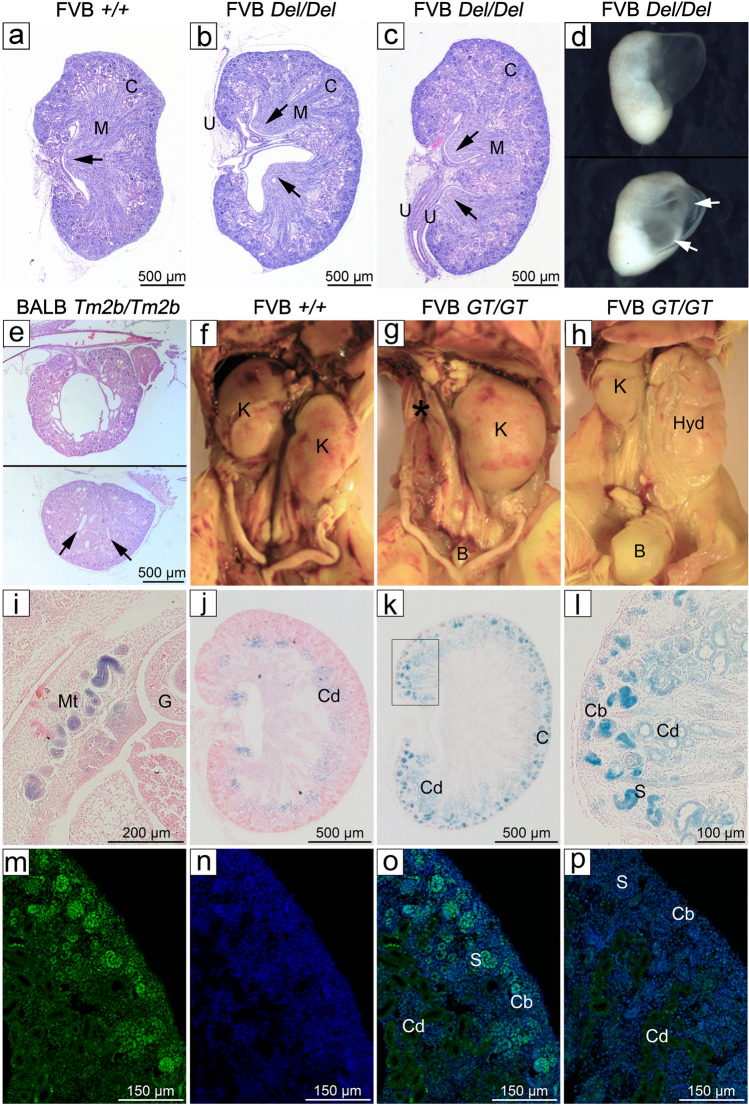
Loss of CECR2 is associated with kidney defects. Coronal sections of E18.5 kidneys show a single renal pelvis (arrow) in a FVB/NJ wild-type control (**a**) and representative duplex kidneys with two renal pelvises in FVB/NJ *Cecr2*^*Del/Del*^ embryos (**b**,**c**). The duplex kidneys ranged from compact (**b**) to elongated (**c**), the latter section which clearly showed two ureters (U). An additional E18.5 FVB/NJ *Cecr2*^*Del/Del*^ embryo showed hydronephrosis, and appeared duplex (arrows) through the fluid-filled cavity in the lower image (**d**). The only kidney defect seen in 27 BALB/cCrlAlt *Cecr2*^*Tm2b/Tm2b*^ embryos was one kidney with a large central cavity, which was likely early hydronephrosis secondary to a duplex kidney (**e**), the latter suggested by what appears to be two pelvises (arrows) in the lower image. Most FVB/NJ *Cecr2*^*GT/GT*^ adults had two normal kidneys (**f**), but 16/168 showed unilateral agenesis (**g**, asterisk) with compensatory hypertrophy of the other kidney. Additionally, 3/168 showed hydronephrosis (**h**, the hydronephrotic right kidney (Hyd) deflated during dissection). The expression of CECR2 in the kidney was determined using X-gal staining of coronal kidney sections from FVB/NJ *Cecr2*^*GT/GT*^ embryos, which contain a LacZ genetrap. Expression was seen as early as E10.5 (**i**) in the mesonephric tubules (Mt). At E18.5 X-gal staining showed non-specific endogenous staining of the collecting ducts (Cd) in a FVB/NJ wild-type control (**j**). In FVB/NJ *Cecr2*^*GT/GT*^ embryos specific X-gal staining was seen in the cortex in the tubules of the forming nephrons: comma bodies (Cb) and S-shaped bodies (S) (**l** is a higher magnification of **k**). The endogenous staining in the collecting ducts was faintly visible. CECR2 protein expression was confirmed by immunofluorescence using a CECR2-specific antibody counterstained with DAPI (**m**–**p**). An E18.5 wild-type BALB/cCrlAlt embryo showed expression in the kidney cortex, specifically in the C and S-shaped bodies and some diffuse staining in the collecting ducts (m-CECR2 antibody, n-DAPI, o-merge). A CECR2 and DAPI merge of a *Cecr2*^*Del/Del*^ kidney showed only non-specific diffuse staining of the collecting ducts. B-bladder, C-kidney cortex, Cb-comma bodies, Cd-collecting tubules, G-gut, Hyd-hydronephrotic kidney, M-kidney medulla, S-shaped bodies, U-ureter.

Kidneys from E18.5 FVB/NJ *Cecr2*^*Del/Del*^ embryos were examined by serial sectioning and H&E staining for structural defects. At least one duplex kidney, with 2 renal pelvises and two ureters, was found in 13/27 mutants (48%) (Fig. 7b,c), compared to a control kidney (Fig. 7a). Four of the 13 mutants showed the duplex phenotype in both kidneys. An additional mutant had an embryonic kidney with evidence of hydronephrosis and appeared to be a duplex (Fig. 7d, sectioning could not be done). Of 9 wild-type littermates examined, all had normal kidneys with 1 renal pelvis (*p* = 0.0136). The kidneys of FVB/NJ *Cecr2*^*GT/GT*^ embryos were also examined, with 8/20 (40%) showing at least 1 duplex kidney (no significant difference, *p* > 0.05, compared to FVB/NJ *Cecr2*^*Del/Del*^ embryos).

While 31% (11/35) of *Cecr2 *^*Del/Del*^ embryos have exencephaly and therefore die at birth^14^, *Cecr2*^*GT/GT*^ embryos show no exencephaly (0/45)^13^, making them ideal to study adult kidneys. In fact, the FVB/NJ the *Cecr2*^*GT*^ mutation was kept as a homozygous line. Unilateral renal agenesis (Fig. 7g) and unilateral hydronephrosis (Fig. 7h) were found in 16/168 (9.5%) and 3/168 (1.7% ) mutant adults examined respectively. Neither was seen in 54 wild-type FVB/NJ animals (Fig. 7f) unilateral renal agenesis *p* = 0.0141, hydronephrosis not significantly different at *p* > 0.05). Pathological examination of 1 hydronephrotic kidney found loss of all medullar and internal structures with a thin and stretched cortex containing crushed glomeruli encasing the single large cyst.

Duplex kidney appears to be only common on the FVB/NJ background. Examining *Cecr2*^*Tm2b/Tm2b*^ embryos on both the C57Bl/6N and BALB/cCrlAlt backgrounds, we found very few kidney defects. For C57Bl/6N *Cecr2*^*Tm2b/Tm2b*^ embryos (E15.5 to E18.5), in 30 mutants only 1 duplex kidney was found. For BALB/cCrlAlt *Cecr2*^*Tm2b/Tm2b*^ embryos, no clearly duplex kidney was seen in 27 mutants, but 1 kidney had a large central cavity which could result from hydronephrosis of a duplex kidney (Fig. 7e).

The role of *Cecr2* in kidney development is further supported by its expression. X-Gal staining shows *Cecr2* is expressed in the tubules of the forming kidney as early as the mesonephros (Fig. 7i). In later development at E18.5 *Cecr2* is expressed in condensing mesenchyme as well as comma and S-shaped bodies of the forming glomeruli (Fig. 7j–l). CECR2 protein expression in forming glomeruli at E18.5 was confirmed using immunofluorescence with a CECR2-specific antibody (Fig. 7m–p).

## Discussion

CES is a rare human disorder with a highly complex and variable phenotype. Because it is usually caused by a 1.5 Mb duplication, it is difficult to study with mouse models. Prior to this report, there was little definitive evidence to suggest which of the at least 14 genes in this duplication contributes to the phenotype, alone or in combination, or whether the smaller duplication of 600 kb containing 3 genes^11^ represents all of the phenotypic abnormalities. Creating a chromosomal duplication in mice is further complicated by the fact that one gene in the human CES critical region, *Cecr1* (aka *Ada2*), is not present in mice^23^. This report now shows that mouse lines with the loss of a single gene in the CES critical region, *Cecr2*, show multiple examples in multiple organs of phenotypic abnormalities reminiscent of those found in human CES, therefore suggesting that *Cecr2* is critical in the pathways leading to those defects in CES.

CES is named after the eye defect coloboma, although only 55–61% of CES patients show this feature^2,3^. We showed that C57Bl/6N *Cecr2*^*Tm2b/Tm2b*^ and *Cecr2*^*Del/Del*^ embryos have a high penetrance of coloboma similar to patients with CES. Microphthalmia, seen in 19–39% of patients with CES^2,3^, was also seen with both presumptive null *Cecr2* mutations on this background, but only associated with coloboma and at a lower penetrance than in humans (8.3–10%). X-gal staining in mice showed that *Cecr2* is expressed at the time of optic fissure closure, supporting its role in this process. We further showed that the presence of this defect in *Cecr2* mutants is dependent on genetic background, with 59–82% penetrance on the C57Bl/6N background but 0% on the BALB/cCrlAlt background. The importance of genetic background mirrors the variability seen in CES. In this study penetrance also appears to be dependent on the mutation, with *Cecr2*^*Tm2b/Tm2b*^ embryos showing significantly higher penetrance (82%) than *Cecr2*^*Del/Del*^ embryos (59%). Although both alleles are presumptive nulls, one possible complication is that the *Cecr2*^*Tm2b*^ mutation was congenic on the C57Bl/6N background (at least 10 generations) while our *Cecr2*^*Del*^ mutation had only been moved from the non-penetrant BALB/cCrlAlt background to the C57Bl/6N background for 3–5 generations. It is possible that remaining BALB/cCrlAlt modifiers affected the coloboma penetrance. A difference in penetrance between the 2 null mutations is not seen with exencephaly (94 vs. 93%), microphthalmia (8.3 vs. 10%) or polydactyly (50 vs. 41%).

Skeletal defects are found in 29–73% of CES cases, depending on the review study^2,3^. We show that *Cecr2* homozygotes can have post- and pre-axial polydactyly, of which only pre-axial involves cartilage or bone. Only one relevant case of CES has been reported with right upper limb post-axial polydactyly^24^. A more common skeletal defect in CES is scoliosis^2,3^. BALB/cCrlAlt *Cecr2*^*GT*^ and *Cecr2*^*Del*^ homozygotes and heterozygotes often show tail kinks, which we showed evidence of being due to malformed vertebrae. These wedge and hemi-vertebrae resemble defects found in human cases of congenital scoliosis^25,26^. It would be interesting to examine *Cecr2* mutant spines to look for subtle changes in the vertebrae.

Heart defects are a major feature of CES (50–63% penetrance)^2,3^, of which 30% of heart defects are total anomalous pulmonary venous drainage (TAPVR) involving abnormal patterning of the pulmonary veins. Although no *Cecr2* homozygous embryos showed classic TAPVR, a high penetrance of a subtle RPV patterning abnormality was seen in mutants but not in controls, regardless of the two genetic backgrounds tested or the *Cecr2* mutation. Two embryos also showed anomalous vessels draining from the right lung into the right superior vena cava, suggestive of partial anomalous pulmonary venous return^20^. These results suggest that *Cecr2* is involved in the patterning of PVs, which are commonly mispatterned in CES. A further 36% of CES heart defects are ventricular septal defects (VSDs)^3^, which were present in 23% C57Bl/6N *Cecr2*^*Tm2b/Tm2b*^ embryos. However, this defect showed strain dependence, since none were present in BALB/cCrlAlt *Cecr2*^*Tm2b/Tm2b*^ embryos. We also observed what appears to be a right aortic arch with isolated origin of the left subclavian artery (LSA) from either the pulmonary trunk or ductus arteriosus. This a rare but previously reported finding in humans^27^. Although it has not been seen in CES, other abnormalities of the great vessels have been. Intriguingly, we have shown that *Cecr2* is expressed in the outflow tract of the heart, which forms the root of the great vessels. Taken together, our data suggests a role for *Cecr2* in the development of the heart, particularly the pulmonary veins.

Kidney defects are seen in 31% of cases with CES^3^. The most common defects are unilateral renal agenesis and hydronephrosis, which are present, although relatively rare, in the FVB/NJ *Cecr2*^*GT/GT*^ adults studied. In embryos there was a high penetrance of duplex kidneys, which have been reported in CES^21,22^. Duplex kidneys are predisposed to hydronephrosis^28^. Unilateral renal agenesis was seen in a significant number of FVB/NJ *Cecr2*^*GT/GT*^ adults (16/168) yet was never seen in E18.5 embryos. It is possible that in a small percentage of mutants, one kidney involutes over time and is no longer detectable in adulthood^29,30^, perhaps due to perinatal hydronephrosis.

Our study shows that *Cecr2* is involved in the development of the eyes, skeleton, heart and kidneys, and that loss of CECR2 results in defects in the organs similar to those seen in CES. We did not study anal anomalies in mice, which are present in 73–81% of cases with CES^2,3^. This would be a useful future study. Furthermore, in order for *Cecr2* to cause these defects through both loss of function and duplication, one would expect the gene to be dosage-sensitive. It was known that *Cecr2* heterozygous embryos have a low penetrance of exencephaly^13^. We have now shown that *Cecr2* heterozygotes also have a low penetrance of coloboma, microphthalmia, tail kinks and polydactyly. This suggests that *Cecr2* is indeed dose-sensitive.

While chromatin remodelling proteins are involved in many processes, including transcription, DNA repair, DNA replication, homologous recombination and chromatin assembly^31^, the specific function of CECR2 and its CERF complex are currently unclear. In human HEK-293T cells, CECR2 has been shown to be involved in DNA double-strand break (DSB) repair^32,33^. However, we recently showed that loss of CECR2 in *Cecr2*^*Del/Del*^ neurospheres does not affect DSB repair^15^. Therefore, while it is unlikely that exencephaly in *Cecr2* mutants is due to a defect in DSB repair, other tissues would need to be tested to determine if the CES-like features involve a loss of DSB repair. We have also previously shown misregulation of mesenchymal/ectodermal transcription factors in *Cecr2*^*GT/GT*^ and *Cecr2*^*Del/Del*^ embryos (Fairbridge et al. 2010). Experiments such as ChIP-Seq would be required to determine whether CECR2 directly or indirectly affects the transcription of specific genes during embryogenesis. Since it is established that CECR2 can be involved in DNA repair in one cell type but not another, determining function with respect to specific abnormal CES phenotypes may require analysis of different embryonic tissues at different stages of development.

Since its first production in 2005^13^, the *Cecr2* mouse has been used to study the lethal neural tube defect exencephaly as a loss of function. The coloboma phenotype that suggests a role for *Cecr2* in CES only appeared when the mutations were crossed onto a C57Bl/6N background, highlighting the importance of strain differences when studying a complex phenotype. Further investigation has revealed skeletal, heart, and kidney defects reminiscent of CES defects, all but polydactyly showing strain dependence. This strain dependency mirrors the variability of CES phenotypes in humans. CES is highly variable, even within families, and this must partially be due to genetic background differences. To see the effect of varying genetic background, one must look at multiple inbred mouse lines, as we see here with *Cecr2* lines. This may be true of other mouse models for other disorders—to see the full phenotype, different backgrounds are needed to mimic the variability in the human population. However, even within inbred strains there is only partial penetrance of the abnormal phenotypes that we saw, suggesting stochastic developmental factors are affecting the outcome as well.

Human reciprocal microdeletion and microduplication syndromes sometimes show some similar phenotypes^34,35^, indicating a need for a tightly controlled dosage of specific gene products to allow normal development. Outside this range, whether the dosage is abnormally high or low, abnormal development may go down similar paths. For instance, the reciprocal 22q11 microdeletion/microduplication syndromes (which do not overlap with the CES region), share features including heart defects, velopharyngeal insufficiency, cleft palate, hearing loss and cognitive deficits^12^, although the penetrance is likely lower in the microduplication syndrome^36^. Reciprocal microdeletion and microduplication of 7q11.23 (the former being Willliams-Beuren syndrome) share features including abnormal brain imaging, heart defects, joint laxity, ADHD, autism and mild cognitive defects^37^. PMP22 is an example of a single gene that produces similar but distinguishable peripheral neuropathies in both a 1.5 Mb microdeletion (Charcot-Marie-Tooth syndrome type 1A, CMT1A) and a reciprocal microduplication (hereditary neuropathy with liability to pressure palsies, HNPP) due to disruption of peripheral nerve myelination^38^.

Based on our observations, we suggest that *CECR2* is involved in many of the abnormal features seen in CES in humans. While a duplication of this gene in mice could give much insight into the syndrome, the *Cecr2* loss of function mutations can also be used to study the features of CES.

## Data Availability

Frozen embryos and sperm from the *Cecr2*^*GT*^ and *Cecr2*^*Del*^ mutations are available from the MMRRC repository. Stock numbers: BALB/cCrlAlt *Cecr2*^*GT*^ (043819-UNC), BALB/cCrlAlt *Cecr2*^*Del*^ (043818-UNC), FVB/NJ *Cecr2*^*GT*^ (043821-UNC), FVB/NJ *Cecr2*^*Del*^ (043820-UNC).
